# Correlation between delayed carpal tunnel syndrome and carpal malalignment after distal radial fracture

**DOI:** 10.1186/s13018-023-03844-z

**Published:** 2023-05-17

**Authors:** Walaa Elwakil

**Affiliations:** Alexandria University, Alexandria, Egypt

**Keywords:** Distal radius, Fracture, Diagnosis, Electrodiagnosis, Wrist, Carpal tunnel syndrome, Nerve entrapment, Carpal malalignment

## Abstract

**Background:**

Delayed carpal tunnel syndrome after Colles’ fracture is a common complication particularly following conservative treatment. The aim of the study was to verify the correlation of different radiological parameters of carpal alignment and the development as well as the severity of DCTS in elderly female patients within 6 months of distal radial fracture (DRF).

**Methods:**

This is a retrospective case–control study that included 60 female patients with DRF within 6 months treated conservatively (30 patients with signs and symptoms suggestive of DCTS and 30 asymptomatic patients as a control group). Electrophysiological evaluation was done for all the participants, as well as radiological assessment to measure parameters of carpal alignment mainly radiocapitate distance (RCD), volar prominence height (VPH) and volar tilt (VT).

**Results:**

There was a statistical significant difference between both groups regarding the radiological parameters of carpal alignment (The mean values of RCD, VT and VPH were − 11.48 mm, − 20.68° angle, and 2.24 mm respectively in the symptomatic group). A strong correlation was found between decrease in the parameters of carpal alignment and the severity of DCTS. Logistic regression analysis showed that VT is strongly involved in the development of DCTS. The threshold value of the VT was − 20.2° angle (sensitivity 0.83; specificity 0.9; odds ratio 45; 95% CI 0.894–0.999; *p* < 0.001).

**Conclusions:**

Anatomical alteration of the carpal tunnel after DRF with dorsal displacement of the carpal bones contribute to the development of DCTS. Decreasing VT and VPH and RCD are the most significant independent predictors for the development of DCTS in conservatively managed DRF.

*Protocol ID*: 0306060.

## Introduction

Carpal tunnel syndrome (CTS) is quite common after a distal radius fracture (DRF) [1, 2]. It may occur at the time of injury (acute) or after several weeks (delayed) [3, 4]. Delayed CTS (DCTS) that develops weeks after DRF is thought to be caused by a change in carpal tunnel anatomy after the fracture heals. Its incidence rate varies between 0.5 and 22% [5]. Fracture malunion, chronically inflammated tenosynovium, volar callus formation, scar formation, and/or offending hardware with subsequent increase in carpal tunnel pressure are among the proposed pathophysiology for delayed CTS following a DRF [2, 3, 6, 7].

Previous studies reported that distal radius malunion, particularly extension of the distal radius has been associated with DCTS especially in patients treated conservatively. Dorsal angulation and distal radius dorsal shift can cause distal carpal row dorsal shift and carpal malalignment [3, 8, 9].

This study aimed to investigate the relationship between changes in carpal alignment and the degree of median nerve entrapment in patients treated conservatively for distal radius fractures through a quantitative analysis of the relative position of the capitate to the radius and electrophysiological evaluation of median nerve. We hypothesized that changes in carpal alignment correlated with the degree of median nerve entrapment in the first 6 months after DRF.

## Methods

The study was carried out on 30 elderly female patients above 60 years with DRF who developed signs and symptoms (either night or day and night symptoms) suggestive of DCTS within 6 months of fracture. Patients were recruited from those attending the outpatient clinic either for rehabilitation or for electrophysiological evaluation.

Exclusion criteria were: (1) patients with clinical signs and symptoms suggestive of carpal tunnel syndrome before the date of fracture, (2) patients with peripheral neuropathy, (3) patients with violent trauma of the upper limb suggestive of direct nerve injury, (4) patients with associated traumatic nerve injury, (5) patients with history of median nerve release, (6) patients with diabetes mellitus, and other metabolic disorders, (7) patients with rheumatological disorders.

History was taken regarding time of onset of the neurological symptoms. The demographic data and anthropometric measures were documented from all the participants [weight, height, and body mass index (BMI)]. All patients in the symptomatic group had paresthesia in the median nerve distribution of the fractured wrist and positive findings in neurological examinations, including the Tinel sign test and Phalen test. CTS-6 score was recorded for each patient to assess the severity of symptoms [10].

For a case–control study design, 30 female patients of matched age and anthropometric measures who did not experience symptoms of CTS on the injured hand up to 6 months after injury were included as controls from among consecutive patients treated conservatively.

A sample of 30 patients in each group was enough to detect the effect of carpal malalignment on the development of DCTS if true at 0.005 alpha error and 0.90 power of the test, assuming an effect size of 1.0.

### Radiological assessment

**Fig. 1:**
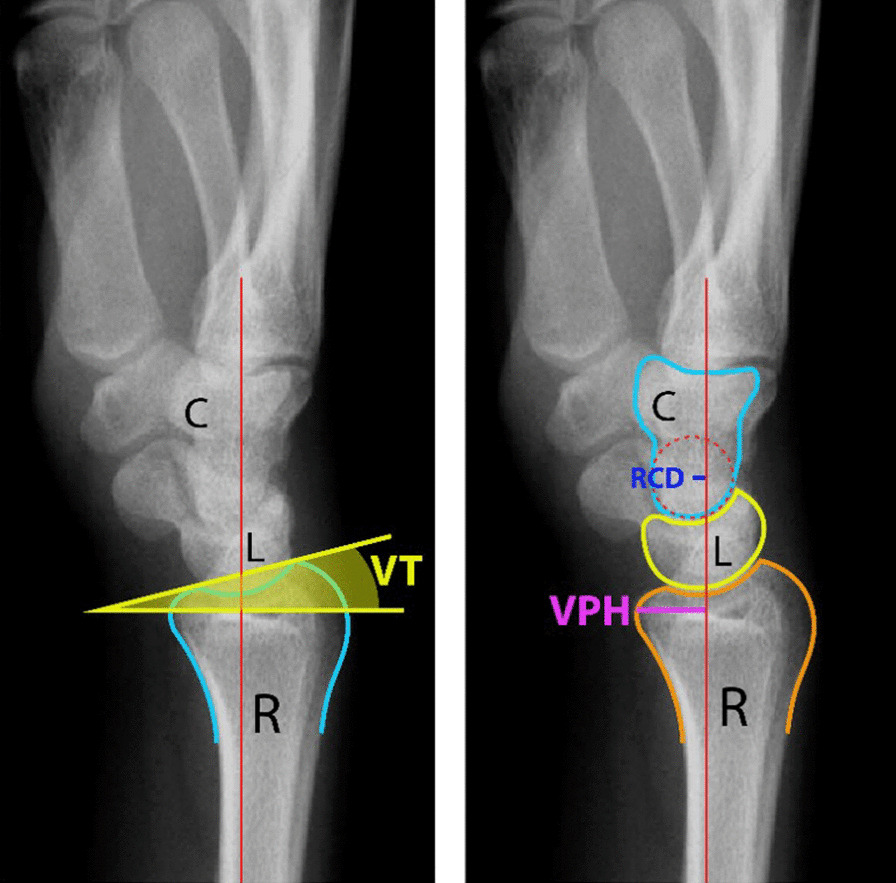
Demonstrating VT, RCD, and VPH. R: radius, L: lunate, C: capitate

Radiological parameters, including the radiocapitate distance (RCD), volar prominence height (VPH), and volar tilt (VT) (Fig. 1), were measured using lateral view radiographs of the wrist at the time of onset of DCTS in the symptomatic group and at 6 months after injury in the control group.

Carpal alignment is typically represented by the radiocarpal and intercarpal angles. The RCD was defined as the distance between the centre of the head of the capitate and the volar cortical line of the radial diaphysis, the VPH was defined as the distance between the vertex of the volar prominence of the distal radial epiphysis and the volar cortical line of the radial diaphysis, and the VT was the angle between the line connecting the dorsal and volar margins of the lunate facet of the distal radius and the line perpendicular to the volar cortical line of the radial diaphysis. A negative VT indicates dorsal angulation of the distal radial articular surface. The RCD is positive when the centre of the capitate's head is on the volar side from the volar cortical line of the radial diaphysis and negative when it is on the dorsal side [11].

### Electrophysiological assessment

The electrophysiological evaluation was done to all the participants. The studies were performed using Viking Quest (Nicolet ViaSys Healthcare, U.S.A.). The following electrophysiological studies were performed: median (APB) and ulnar (ADM) motor nerve conduction studies and median (index) and ulnar (little finger) sensory nerve conduction studies. Median versus ulnar comparative studies were done if there was no electrophysiological abnormality in the median sensory conduction study. The classification of neurophysiological severity of median entrapment across the wrist was according to the Padua neurophysiological classification scale [12].

### Statistical analysis

Data were fed to the computer and analyzed using IBM SPSS software package version 20.0 **(**Armonk, NY: IBM Corp**)**. Student t-test was used to compare both groups regarding the radiological parameters. Correlations between CST-6 score, Padua neurophysiological classification scale for CTS and the different radiological parameters were examined in the DCTS group. The threshold value and the odds ratio of each radiological parameter were examined using univariate logistic regression analysis. Significance of the obtained results was judged at the 5% level.

## Results

The study included 60 female patients with a history of DRF in the last 6 months. Half of them had signs and symptoms suggestive of DCTS and constitute the symptomatic group and the other half represent the control group. Both groups were comparable regarding age and body mass index (BMI) (Table 1).The mean period from injury to the onset of DCTS in the symptomatic group was 3 months (range between 7 weeks and 6 months), and the mean CTS- 6 score in the symptomatic group was 19.3 (range between 17 and 22).

**Table 1:** Comparison between the two studied groups according to age and BMI

	Cases (*n* = 30)	Control (*n* = 30)	Test of sig	*p*
*Age (years)*				
Min.–Max	60–75	60–77	*t* = 0.028	0.978
Mean ± SD	66.9 ± 4.05	67 ± 5.09		
Median (IQR)	67.5 (63–70)	66.5 (62–70)		
*BMI (kg/m*^*2*^*)*				
Min.–Max	21.5–42.0	21.5–39.5	*U* = 1.121	0.267
Mean ± SD	31.2 ± 4.96	29.8 ± 4.90		
Median (IQR)	31 (26.7–34.9)	29.6 (26.7–33.3)		

*IQR* Inter quartile range, *SD* Standard deviation, *χ*^*2*^ Chi square test, *t* Student *t*-test, *U* Mann Whitney test

*p*: *p* value for comparing between the two studied groups

Radiological parameters were measured in both groups (Table 2). The mean RCD, VPH and VT in the symptomatic group were − 11.48 mm, 2.24 mm, and − 20.68° angle, respectively. All these parameters were significantly lower in the symptomatic group compared to the control group.

**Table 2:** Comparison between the two studied groups according to different radiological parameters and electrophysiological findings

	Cases (*n* = 30)	Control (*n* = 30)	Test of sig	*p*
*Radiocapitate distance*				
Min.–Max	− 15.70 to − 5.60	− 10.20 to − 4.50	*U* = 76.0*	< 0.001*
Mean ± SD	− 11.48 ± 2.24	− 7.45 ± 1.95		
Median (IQR)	− 11.25 (− 12.9 to − 10.8)	− 6.80 (− 10.1 to − 5.9)		
*Volar tilt*				
Min. – Max	− 25.50 to − 4.70	− 20.40 to − 1.70	*U* = 48.0*	< 0.001*
Mean ± SD	− 20.68 ± 5.45	− 8.57 ± 7.34		
Median (IQR)	− 22.15 (− 23.2 to − 20.9)	− 4.85 (− 18.2 to − 3.5)		
*Volar prominence height*				
Min.–Max	0.20–5.70	2.80–8.80	*U* = 80.0*	< 0.001*
Mean ± SD	2.24 ± 1.35	4.83 ± 1.75		
Median (IQR)	2.15 (1.40–3.0)	4.35 (3.4–5.3)		
*Padua neurophysiological classification scale for CTS*				
1	0 (0.0%)	28 (93.3%)	*χ*^2^ = 63.078*	^MC^*p* < 0.001*
2	8 (26.7%)	2 (6.7%)		
3	14 (46.7%)	0 (0.0%)		
4	7 (23.3%)	0 (0.0%)		
5	1 (3.3%)	0 (0.0%)		

*IQR* Inter quartile range, *SD* Standard deviation, *t* Student *t*-test, *χ*^*2*^ Chi square test, *MC* Monte Carlo, *U* Mann Whitney test

*p*: *p* value for comparing between the two studied groups

*Statistically significant at *p* ≤ 0.05

An electrophysiological evaluation was done for all the patients (Table 2). Among the symptomatic group about 46% (17 patients) had mild CTS, 23% had moderate CTS and one patient had CTS of severe degree, while in the control group only 2 patients had abnormal comparative studies (minimal CTS).

In the symptomatic group; a significant negative correlation was reported between the clinical assessment score CTS-6 as well as the degree of severity of CTS according to Padua neurophysiological classification scale for CTS and the radiological parameters of distal radial displacement; RCD, VT and VPH (Table 3).

**Table 3:** Correlation between and different parameters in cases group

Cases (*n* = 30)	Padua neurophysiological classification scale for CTS		CTS-6 assessment score	
	*r*_s_	*p*	*r*_s_	*p*
Volar tilt	− 0.729*	< 0.001*	− 0.656*	< 0.001*
Volar prominence height	− 0.678*	< 0.001*	− 0.642*	< 0.001*
Radiocapitate distance	− 0.711*	< 0.001*	− 0.610*	< 0.001*

*r*_s_: Spearman coefficient

*Statistically significant at *p* ≤ 0.05

**Fig. 2:**
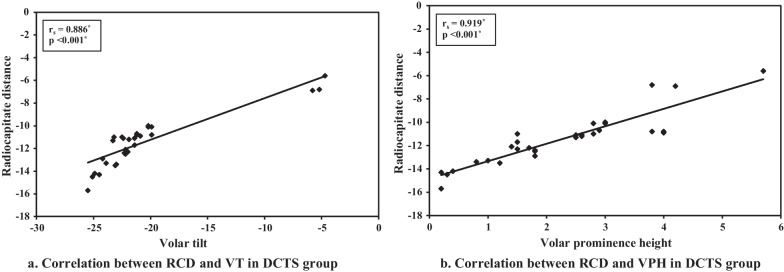
**a** Correlation between RCD and VT in DCTS group **b** Correlation between RCD and VPH in DCTS group

**Fig. 3:**
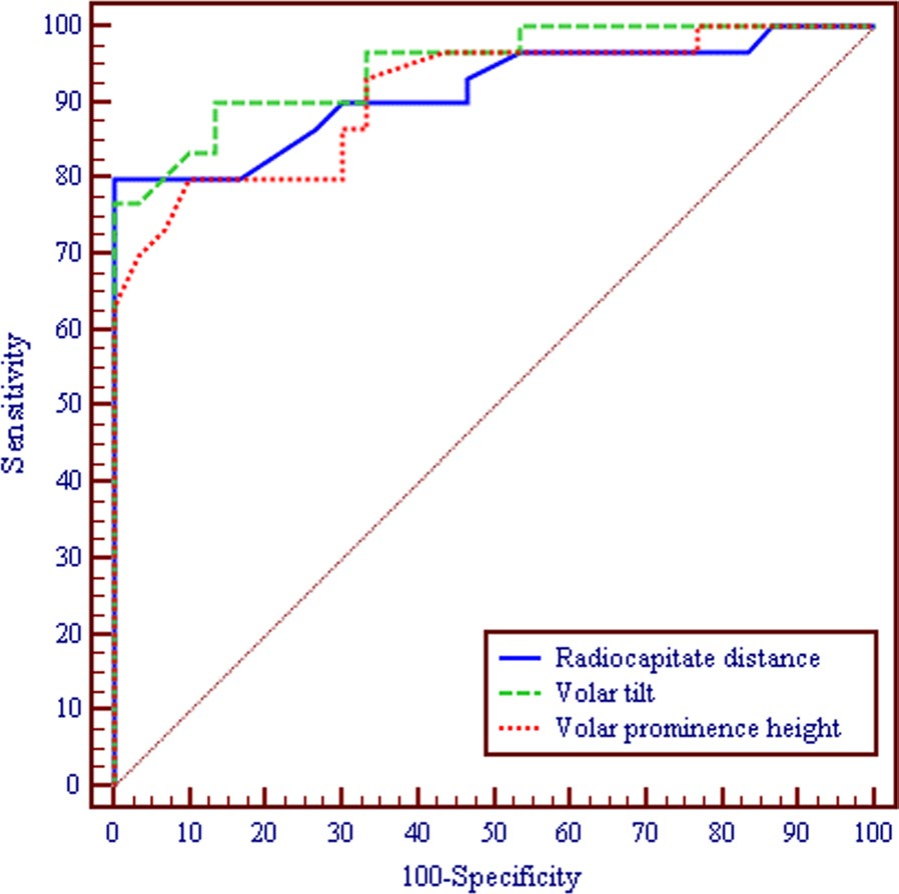
3ROC curves for RCD, VT and VPH

Figure 2 demonstrates a strong correlation between RCT, VT and VPH in the symptomatic group. Tables 4 and 5 represent the results of logistic regression analysis and the odds ratio of each radiological parameter. According to the ROC curves (Fig. 3), the threshold value of the RCD was − 10.2 mm (sensitivity, 0.8; specificity, 0.83; odds ratio, 20; 95% CI 0.840–0.991; *p* < 0.001). The threshold value of the VT − 20.2° angle (sensitivity, 0.83; specificity, 0.9; odds ratio, 45; 95% CI 0.894–0.999; *p* < 0.001). Regarding the VPH the threshold value was 3 mm (sensitivity, 0.8; specificity, 0.9; odds ratio, 36; 95% CI 0.838–0.984; *p* < 0.001).

**Table 4:** Validity (AUC, sensitivity, specificity) for the radiological parameters to diagnose patients (*n* = 30) from control (*n* = 30)

	AUC	*p*	95% CI	Cut off	Sensitivity	Specificity	PPV	NPV
Radiocapitate distance	0.916*	< 0.001*	0.840–0.991	≤ − 10.2	80.0	83.33	82.8	80.6
Volar tilt	0.947*	< 0.001*	0.894–0.999	≤ − 17.2	83.33	90.0	89.3	84.4
Volar prominence height	0.911*	< 0.001*	0.838–0.984	≤ 3	80.0	90.0	88.9	81.0

*AUC* Area under a curve, *p value* Probability value, *CI* Confidence Intervals, *NPV* Negative predictive value, *PPV* Positive predictive value

*Statistically significant at *p* ≤ 0.05

**Table 5:** Results of univariate logistic regression analysis of each radiological parameter

	OR	95% CI	*p*
Radiocapitate distance (≤ − 10.2)	20.0*	5.384–74.298	< 0.001*
Volar tilt (≤ − 20.2)	45.0*	9.732–208.07	< 0.001*
Volar prominence height (≤ 3)	36.0*	8.105–159.89	< 0.001*

*OR* Odd’s ratio, *CI* Confidence interval

*Statistically significant at *p* ≤ 0.05

## Discussion

The aetiology of CTS after a DRF is likely multifactorial and has been linked to changes in carpal tunnel anatomy after the traumatic event [3, 13, 14]. According to Itsubo et al. [9]. DCTS was defined as CTS occurring > 12 weeks after injury. According to our results the mean period from injury to the onset of DCTS in the symptomatic group was 3 months. When compared to the control group, all the radiological parameters of distal radius alignment were significantly lower in the symptomatic group.

Many authors have speculated ideas between malalignment of DRF and development of DCTS. Itsubo et al. [9] examined 30 cases of DCTS and concluded that fracture malalignment resulting in changes in the anatomical configuration of the carpal tunnel could be a risk factor for the development of DCTS. Stewart et al. [8] found that the VT was significantly lower in cases with DCTS compared to cases without CTS (− 12.6° angle versus − 7.0° angle). Kwasny et al. [15] reported that the VT less than − 20° angle was associated with increased risk of DCTS.

Recently, Watanabe and Ota [16] mentioned that the mean VT was significantly lower in the DCTS group relative to the control group (− 20.5° angle versus − 11.3° angle) and reported that carpal malalignment, specifically dorsal displacement of capitate, resulting from malunion of the distal radius is a predictor of DCTS, and considered the RCD as the major factor involved in the development of DCTS. On the other hand, our results reported that VT was even more predictive with an odds ratio of 45. This was in agreement with the study conducted by Kim et al. [17] in which VT and tear drop angle (TDA) were significant independent predictors of development of DCTS.

Adding the electrophysiological evaluation for all the patients was valuable to support the clinical diagnosis of CTS with objective data. In the current study all the symptomatic patients had electrophysiological features suggestive of CTS in addition to 6.6% of the asymptomatic group. Stark et al. [18] reported that electrodiagnostic studies were “abnormal” in 52.5% of fractured hands, with only half of them had documented paresthesia, dysesthesia, or sensory impairment on testing. Moreover, there is no report about the relationship between radiological parameters of DRF malalignment and the degree of severity of CTS according to the electrophysiological studies. The present study found a significant negative correlation between the degree of severity of CTS and the different radiological parameters of distal radial malalignment.

This study had some limitations. The sample size was small, with only female patients over the age of 60 included. Although the results are highly significant, a larger sample size study with both sexes and different age groups is recommended. Additionally, it is worth comparing the values of the radiological parameters between the fractured and sound sides, especially in small-series studies. Furthermore, other radiological parameters that seem to be reproducible, such as ulnar variance, radial inclination, scapholunate angle (SLA), capitolunate angle (CLA), radiolunate angle (RLA), and tear drop angle (TDA) were not measured in the current study.

A future study on the symptomatic group, supplemented by ultrasound evaluation of median nerve and carpal tunnel structures, could provide a clear insight into the nature and common sites of median nerve involvement after DRF, as well as the altered flexor tendon mechanisms that contribute to increased pressure under the transverse carpal ligament.

## Conclusions

DCTS can appear weeks or months after a DRF. A better understanding of the risk factors for DCTS can guide surgeons' decision-making regarding the management of DRF in order to enhance neurological recovery and to improve functional outcomes. Decreased volar tilt, volar prominence height, and radiocapitate distance are independent predictors for the development of DCTS after DRF.

## Data Availability

The datasets analyzed during the current study are available from the corresponding author on reasonable request.

## Abbreviations

ADM: Abductor digiti minimi
APB: Abductor poliicis brevis
BMI: Body mass index
CLA: Capitolunate angle
CTS: Carpal tunnel syndrome
DCTS: Delayed carpal tunnel syndrome
DRF: Distal radius fracture
OR: Odds ratio
RCD: Radiocapitate distance
RLA: Radiolunate angle
ROC: Receiver operating characteristic
SLA: Scapholunate angle
TDA: Tear drop angle
VT: Volar tilt
VPH: Volar prominence height
